# Establishing an enzyme cascade for one-pot production of α-olefins from low-cost triglycerides and oils without exogenous H_2_O_2_ addition

**DOI:** 10.1186/s13068-020-01684-1

**Published:** 2020-03-16

**Authors:** Yuanyuan Jiang, Zhong Li, Shanmin Zheng, Huifang Xu, Yongjin J. Zhou, Zhengquan Gao, Chunxiao Meng, Shengying Li

**Affiliations:** 1 Shandong Provincial Key Laboratory of Synthetic Biology, CAS Key Laboratory of Biofuels, Qingdao Institute of Bioenergy and Bioprocess Technology, Chinese Academy of Sciences, No. 189 Songling Road, Qingdao, 266101 Shandong China; 2 University of Chinese Academy of Sciences, Beijing, 100049 China; 3 State Key Laboratory of Microbial Technology, Shandong University, Qingdao, 266237 Shandong China; 4 School of Life Sciences, Shandong University of Technology, Zibo, 255000 Shandong China; 5 Division of Biotechnology, Dalian Institute of Chemical Physics, Chinese Academy of Sciences, Dalian, 116023 Liaoning China; 6 Laboratory for Marine Biology and Biotechnology, Qingdao National Laboratory for Marine Science and Technology, Qingdao, 266237 Shandong China

**Keywords:** Lipase, P450 fatty acid decarboxylase, Alditol oxidase, Bio-catalysis, Triglycerides, Natural oils, α-Olefins

## Abstract

**Background:**

Biological α-olefins can be used as both biofuels and high value-added chemical precursors to lubricants, polymers, and detergents. The prototypic CYP152 peroxygenase family member OleT_JE_ from *Jeotgalicoccus* sp. ATCC 8456 catalyzes a single-step decarboxylation of free fatty acids (FFAs) to form α-olefins using H_2_O_2_ as a cofactor, thus attracting much attention since its discovery. To improve the productivity of α-olefins, significant efforts on protein engineering, electron donor engineering, and metabolic engineering of OleT_JE_ have been made. However, little success has been achieved in obtaining α-olefin high-producer microorganisms due to multiple reasons such as the tight regulation of FFA biosynthesis, the difficulty of manipulating multi-enzyme metabolic network, and the poor catalytic performance of OleT_JE_.

**Results:**

In this study, a novel enzyme cascade was developed for one-pot production of α-olefins from low-cost triacylglycerols (TAGs) and natural oils without exogenous H_2_O_2_ addition. This artificial biocatalytic route consists of a lipase (CRL, AOL or Lip2) for TAG hydrolysis to produce glycerol and free fatty acids (FFAs), an alditol oxidase (AldO) for H_2_O_2_ generation upon glycerol oxidation, and the P450 fatty acid decarboxylase OleT_JE_ for FFA decarboxylation using H_2_O_2_ generated in situ. The multi-enzyme system was systematically optimized leading to the production of α-olefins with the conversion rates ranging from 37.2 to 68.5%. Furthermore, a reaction using lyophilized CRL/OleT_JE_/AldO enzymes at an optimized ratio (5 U/6 μM/30 μM) gave a promising α-olefin yield of 0.53 g/L from 1500 μM (~1 g/L) coconut oil.

**Conclusions:**

The one-pot enzyme cascade was successfully established and applied to prepare high value-added α-olefins from low-cost and renewable TAGs/natural oils. This system is independent of exogenous addition of H_2_O_2_, thus not only circumventing the detrimental effect of H_2_O_2_ on the stability and activity of involved enzymes, but also lower the overall costs on the TAG-to-olefin transformation. It is anticipated that this biotransformation system will become industrially relevant in the future upon more engineering efforts based on this proof-of-concept work.

## Background

Volatile geopolitical factors, depletion of petroleum-based fuels, and serious environmental concerns have been spurring the development of alternative, sustainable, and cost-effective biofuels from renewable feedstocks [1, 2]. Biofuels are considered as the most promising green alternatives to petroleum-based fuels because their combustion could be carbon neutral or even negative (when produced by photosynthetic microalgae) and of near-zero air pollution [3]. Among different biofuel types, aliphatic hydrocarbons such as fatty alkanes and alkenes are regarded as ideal biofuels due to their high energy content, low hygroscopicity, and compatibility with the existing engine and distribution systems [4, 5].

Chemically, a number of thermochemical methods including gasification, pyrolysis, and liquefaction, and various metal-based catalytic reactions can be utilized to convert biomass into hydrocarbon-based fuels [6, 7]. However, these chemical approaches often lead to poor hydrocarbon yields, high energy consumption, and considerable side products due to simultaneous occurrence of several types of undesired reactions at high temperatures. Thus, these problems together with significant environmental concerns on hazardous chemical wastes have prompted continuous searches for green, robust, and economic biocatalytic methods to produce aliphatic hydrocarbons [8, 9].

Compared to fatty alkanes, fatty alkenes especially terminal olefins (i.e., α-olefins) are more valuable products since they can be used as both biofuels and important precursors to lubricants, polymers, and detergents [10]. Nature has evolved various α-olefin-producing enzymes that use either free fatty acids (FFAs) or fatty acyl–acyl carrier proteins (acyl-ACPs) as starting materials [8]. Among them, P450 fatty acid decarboxylases have attracted the most attention in recent years because this P450 enzyme family efficiently catalyzes a single-step decarboxylation of FFAs to form α-olefins by consuming H_2_O_2_ (as sole oxygen and electron donor) stoichiometrically, with varying amounts of hydroxylated fatty acids as side products.

As the first identified P450 fatty acid decarboxylase, OleT_JE_ from *Jeotgalicoccus* sp. ATCC 8456 [11] has been intensively studied to understand its unique catalytic mechanism and to harness its valuable decarboxylation capacity [12–17]. Furthermore, significant efforts on protein engineering, electron donor engineering, pathway engineering, and metabolic engineering have been made for both titer improvement and product profile tuning [14, 18, 19]. Nonetheless, the reported highest total alkene titers of different engineered microorganisms have only reached the level of several 100 mg/L, which are far from the high cost requirement of commercial production of α-olefins. The major reasons accounting for the low α-olefin yields include the tightly regulated FFA biosynthesis from glucose, the difficulty of manipulating multi-enzyme metabolic network, and the poor catalytic behaviors of OleT_JE_ likely owing to poor stability, low activity, substrate accessibility, and the availability of H_2_O_2_ cofactor in vivo [18, 20, 21].

Thus, the multi-enzyme in vitro transformation of renewable oil-based feedstock into α-olefins has appeared to be a promising alternative strategy. For example, in our previous study, a two-enzyme system consisting of the lipase Tll for hydrolysis of triglycerides (TAGs) to generate FFAs and OleT_JE_ for decarboxylation of the resulting FFAs to produce α-olefins in the presence of exogenously added H_2_O_2_ was engineered; and the overall TAG-to-olefin yields reached 6.7–46.0% [19]. Matthews et al. recently engineered an OleT_JE_–AldO fusion enzyme, in which the alditol oxidase AldO from *Streptomyces coelicolor* [22] was responsible for oxidizing glycerol to glyceraldehyde and glyceric acid sequentially and generating H_2_O_2_ as a co-product to drive the following OleT_JE_-mediated FFA decarboxylation.

**Fig. 1:**
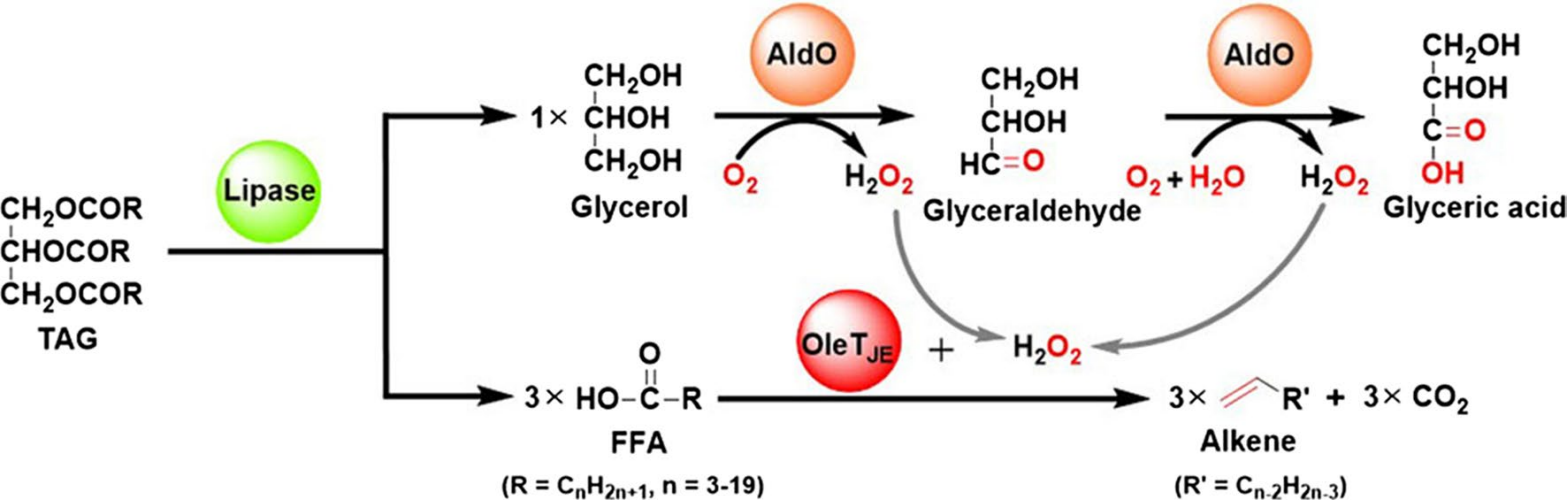
The schematic enzyme cascade for one-pot production of α-olefins from low-cost triglycerides

To further improve the efficiency of TAG-to-olefin transformation and lower the overall costs, a number of key factors including the catalytic efficiencies, the mismatch between the fatty acyl chain length specificity of lipase and P450 fatty acid decarboxylase, the cofactor supply, and the low stability of proteins (especially for P450 enzymes) in presence of H_2_O_2_ must be addressed. Thus, in this work, a novel enzyme cascade was designed and assembled for efficient one-pot production of α-olefins from low-cost triglycerides and natural oils by integrating the activities of lipase, P450 fatty acid decarboxylase, and alditol oxidase. Conceptually, a lipase hydrolyzes one molecule of TAG to release three molecules of FFAs and one molecule of glycerol; an alditol oxidase (AldO) [22] oxidizes glycerol to generate two equivalents of H_2_O_2_ in situ as the cofactor of OleT_JE_ to drive FFA decarboxylation yielding *α*-olefins; the shortage of one equivalent of H_2_O_2_ can be resolved by exogenous addition of glycerol (Fig. 1).

In specific, three well-characterized lipases including CRL from *Candida rugose* [23], AOL from *Aspergillus oryzae* [24], and Lip2 from *Yarrowia lipolytica* [25] were examined for their cooperativity with OleT_JE_ and AldO toward different substrates, including pure TAGs, and several kinds of natural oils. Moreover, the enzyme cascade comprising CRL, OleT_JE_, and AldO was optimized for the preferable coconut oil using lyophilized enzymes.

## Results

### Lipase selection in terms of the TAG hydrolytic activity

**Fig. 2:**
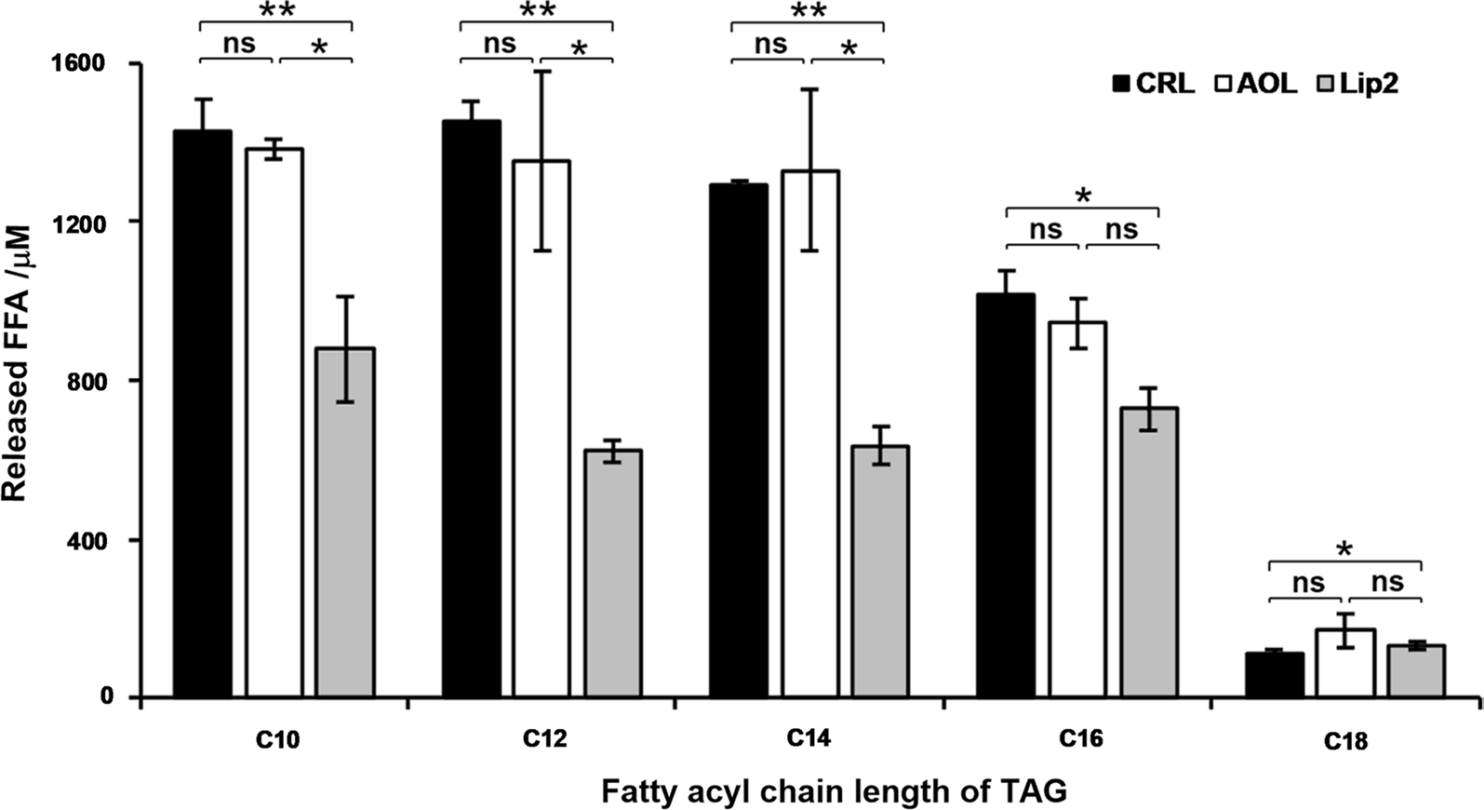
Released FFAs from 500 μM of different pure TAGs by CRL, AOL, and Lip2. In a standard assay, 5 U lipase and 500 μM TAG were co-incubated in a 200-μL reaction system at 30 °C for 6 h. Error bars represent standard deviations derived from at least two independent experiments. Statistical analysis was performed using a Student’s *t* test (one-tailed; *P < 0.05, **P < 0.01, ns: *P *> 0.05, no significant; two-sample unequal variance) (same below)

The P450 fatty acid decarboxylase OleT_JE_ favors the FFAs with the acyl chain length between C_10_ and C_18_ [18, 26]. To identify an appropriate lipase to efficiently supply the favorable FFA substrates for OleT_JE_, the hydrolytic activities of three well-characterized lipases toward the pure TAGs (500 μM) with the fatty acyl chains of C_10_–C_18_ were evaluated, including the two commercial lipases, namely, CRL from *Candida rugose* and AOL from *Aspergillus oryzae*, and the laboratory-prepared Lip2 from *Yarrowia lipolytica* (Additional file 1: Figure S1) [23–25]. As results, CRL released 1429.9 μM capric acid (C_10_), 1450.8 μM lauric acid (C_12_), 1293.6 μM myristic acid (C_14_), 1016.8 μM palmitic acid (C_16_), and 109.6 μM stearic acid (C_18_) from their corresponding TAGs, corresponding to the conversion rates of 95.3%, 96.7%, 86.2%, 67.8%, and 7.3%, respectively (Fig. 2). AOL exhibited analogous hydrolytic activities and a similar substrate preference profile to CRL with tricaprin (C_10_) as the optimal substrate (92.3% conversion rate). However, the laboratory-prepared Lip2 showed significantly lower activities than both AOL and CRL, with the highest yield of 58.6% against tricaprin (C_10_). Considering the overall hydrolytic activity and the factor that OleT_JE_ prefers C_10_–C_14_ FFAs [26], CRL and AOL were selected for the following enzyme cascade setup.

### Effect of H_2_O_2_ concentration on the OleT_JE_ activity

OleT_JE_, a CYP152 peroxygenase family member, utilizes H_2_O_2_ as a cofactor (or co-substrate) to support the unique oxidative decarboxylation reactions that convert C_*n*_ (*n* = 4–22) chain length FFAs into C_*n*–1_ α-olefins and release one molecule of CO_2_ [26]. In principle, a higher concentration of H_2_O_2_ co-substrate presumably enhances the reaction rate kinetically. However, excessive addition of H_2_O_2_ would oxidatively inactivate enzymes via radical mechanisms in general [27]. Therefore, it is important to balance the two opposite sides of H_2_O_2_ by identifying an optimal working concentration of H_2_O_2_ for OleT_JE_’s activity.

**Fig. 3:**
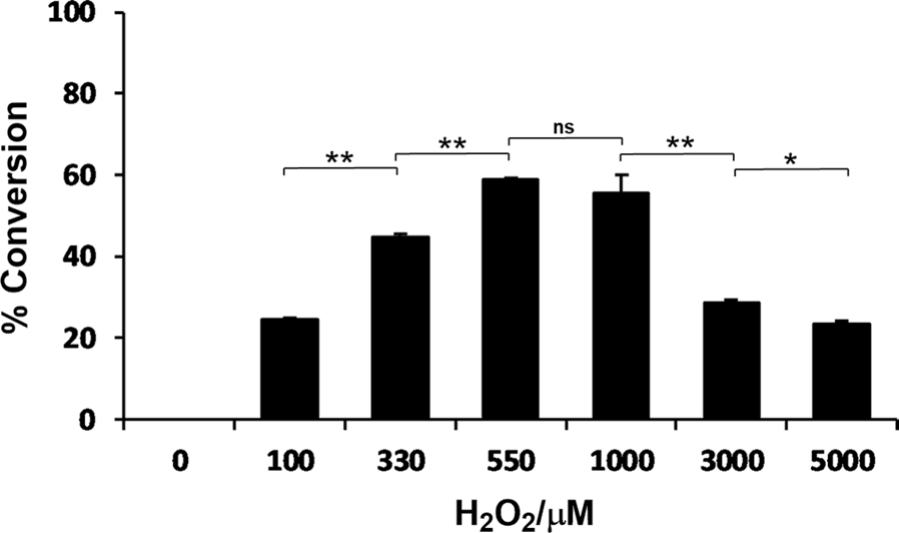
Effect of H_2_O_2_ concentration on the OleT_JE_ activity

In specific, 0–5000 μM of H_2_O_2_ was added into each individual OleT_JE_ reaction mixture containing 1 μM P450 enzyme and 500 μM lauric acid. As expected, the conversion of lauric acid increased in proportion to the amount of H_2_O_2_ at the low concentration range (0–550 μM); then, the substrate conversion rates declined with the increase of H_2_O_2_ concentrations between 1000 and 5000 μM (Fig. 3). Taken together, direct addition of H_2_O_2_ to a high concentration appeared not to be an effective way to maintain a high activity of OleT_JE_.

### Determination of the optimal ratio between OleT_JE_ and AldO

Although the OleT_JE_–AldO fusion enzyme developed by Munro and co-workers could enable efficient channeling of H_2_O_2_ between these two enzyme active sites [28], the fusion nature of OleT_JE_ and AldO fixed the molar ratio of these two functional domains to be 1:1, which might not be an optimal ratio. Moreover, the chimeric protein might cost its high expression level.

**Fig. 4:**
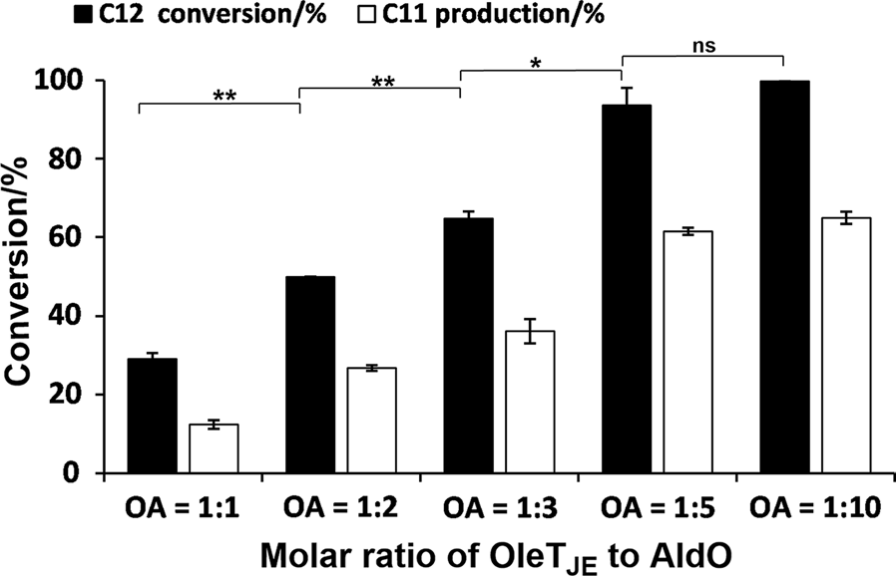
Effect of the molar ratio of OleT_JE_ to AldO (OA) on the OleT_JE_ activity at a low glycerol loading (0.01% *v/v*)

Thus, in this study, OleT_JE_ and AldO were separately expressed (Additional file 1: Figure S2), by which their ratio could be conveniently adjusted. Using 500 μM lauric acid as substrate and 0.01% *v/v* (~ 1000 μM) glycerol loading, a select number of different OleT_JE_ to AldO (OA) ratios were examined. It was revealed that the best OA ratio turned out to be 1:10 (or 1:5), at which the substrate conversion rate and the alkene production ratio were 99.7% (93.6%) and 64.9% (61.5%) (Fig. 4), respectively. The differences between lauric acid (C_12_) conversion and undecene (C_11_) production were due to the unquantified side products, namely, different hydroxylated fatty acids. In consideration of both the overall catalytic efficiency and cost-effectiveness, the OA ratio of 1:5 with the lower amount of AldO was selected for the following experiments.

### Determination of the optimal glycerol concentration

As the substrate of AldO, glycerol is presumably a key factor in the OleT_JE_/AldO reaction system. Thus, the optimal glycerol concentration for OleT_JE_’s activity in the range of 0.01–10% was determined with the exogenous addition of H_2_O_2_ as a control. For 500 μM lauric acid, 0.01% glycerol loading resulted in approximately 100% increase in both substrate conversion rate and total turnover number (TTN) compared to the OleT_JE_ reaction supported by 500 μM H_2_O_2_ (Table 1). These results clearly demonstrated the advantage of continuous release of H_2_O_2_ over the direct addition of H_2_O_2_ at once.

**Table 1:** OleT_JE_ catalytic activities in two different H_2_O_2_-supplying systems

Substrate	H_2_O_2_ supply	Conversion/%^a^	TTN^a^
500 μM lauric acid	500 μM H_2_O_2_	46.5	235 ± 4
	AldO + 0.01% glycerol	93.6	467 ± 22**
1000 μM lauric acid	1000 μM H_2_O_2_	18.1	181 ± 2
	AldO + 0.01% glycerol	27.3	273 ± 4*
	AldO + 0.05% glycerol	35.4	354 ± 10*
	AldO + 0.1% glycerol	38.6	386 ± 31^ns^
	AldO + 0.5% glycerol	49.3	493 ± 24*
	AldO + 1% glycerol	50.1	501 ± 39*
	AldO + 5% glycerol	64.9	649 ± 23*
	AldO + 10% glycerol	69.5	695 ± 40^ns^

^a^Reaction conditions: 1 μM OleT_JE_ supported by H_2_O_2_ or the 5 μM AldO + glycerol system, at 30 °C for 6 h. Total turnover number (TTN): mean ± standard deviation; standard deviations were derived from at least two independent experiments. Statistical analysis of the adjacent TTN data was performed using a Student’s *t* test (one-tailed; *P < 0.05, **P < 0.01, ns: *P *> 0.05, no significant; two-sample unequal variance)

At a higher concentration of lauric acid (1000 μM), the AldO-based in situ H_2_O_2_-generating system showed more significant improvements under all tested glycerol concentrations relative to 1000 μM H_2_O_2_ (Table 1). In the presence of 0.01–10% glycerol, the TTNs ranged from 273 to 695. When the glycerol concentration was higher, the decarboxylation activity was better. Surprisingly, at the same 0.01% glycerol loading, the reaction with 1000 μM substrate gave a much lower TTN than that with 500 μM substrate. This result suggested that the activity of AldO might be inhibited by the high concentration of hydrophobic products or substrates at a relatively low glycerol concentration.

Evidently, 1 μM OleT_JE_ and 5 μM AldO were not sufficient for complete decarboxylation of 1000 μM lauric acid even in the presence of 10% glycerol. To decarboxylate 1500 μM lauric acid that can be maximally released from 500 μM trilaurin, higher concentrations of OleT_JE_ and AldO were required to be used with an appropriate amount of glycerol. Thus, the reaction system of 3 μM OleT_JE_, 15 μM AldO, and 1.5% glycerol was proposed and tested, by which we assumed that a triplicated TTN could be achieved by higher enzyme concentrations.

### Establishing an enzyme cascade for the transformation from TAGs to α-olefins without exogenous H_2_O_2_ addition

**Fig. 5:**
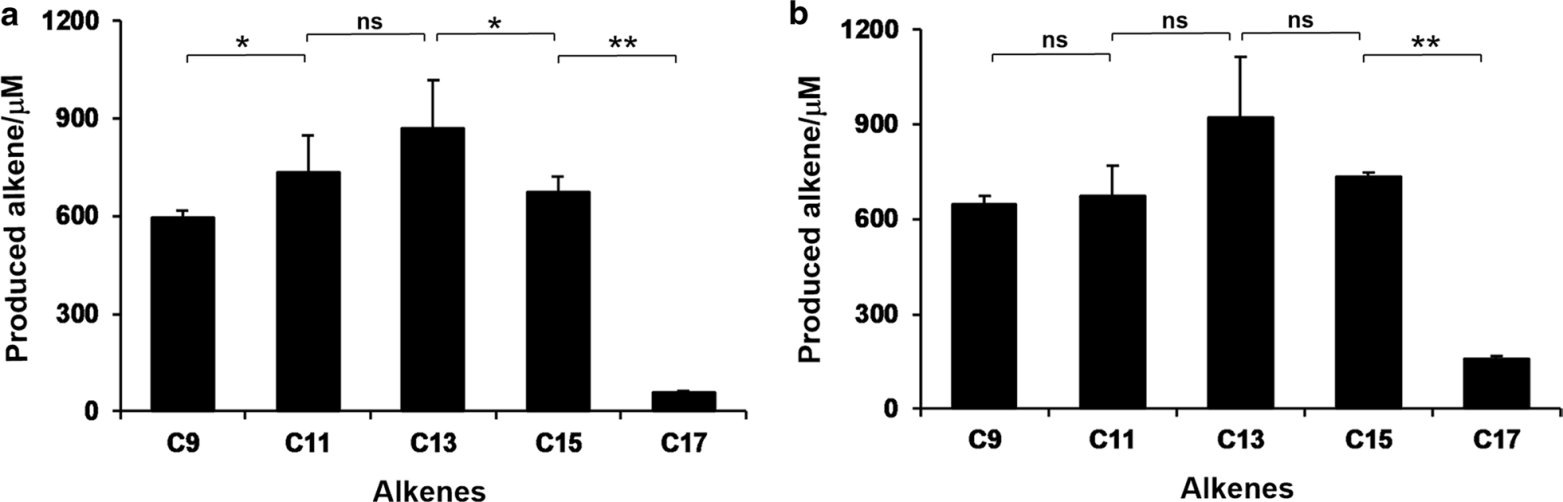
Production of α-alkenes from TAGs by the lipase/OleT_JE_ cascade supported by AldO/glycerol. **a** CRL/OleT_JE_/AldO and **b** AOL/OleT_JE_/AldO

Previously, an enzyme cascade was established in this laboratory comprising a lipase and a P450 fatty acid decarboxylase, which is capable of converting TAGs into α-olefins with exogenous addition of H_2_O_2_ [19]. To make this system independent of detrimental exogenous H_2_O_2_, AldO was introduced into this enzyme cascade to generate H_2_O_2_ in situ from glycerol, the by-product of lipase. In specific, 3 μM OleT_JE_, 15 μM AldO, 5 U lipase, 1.5% glycerol, and 500 μM pure TAGs were mixed together and incubated at 30 °C for 6 h. As results, 594.7 μM 1-nonene (C_9_), 735.5 μM 1-undecene (C_11_), 871.4 μM 1-tridecene (C_13_), 673.3 μM 1-pentadecene (C_15_), and 56.4 μM 1-heptadecene (C_17_) were produced from respective TAGs by the CRL/OleT_JE_/AldO system (Fig. 5a), corresponding to 39.6%, 49.1%, 58.1%, 44.9%, and 3.8% of the theoretical maximum TAG-to-olefin yields (TAG:FFA:*α*-olefin = 1:3:3). The reactivity pattern of the AOL/OleT_JE_/AldO was similar to that of the CRL/OleT_JE_/AldO system (Fig. 5), while the yields of 1-tridecene (61.5%) and 1-heptadecene (10.6%) reached a higher level.

### Production of α-olefins from natural oils

Next, the activities of the CRL/OleT_JE_/AldO and AOL/OleT_JE_/AldO systems were examined toward a number of natural oils including coconut oil and palm oil featuring saturated fats, peanut oil and olive oil containing monounsaturated fats, and soybean oil representing polyunsaturated fats. Compared to pure TAGs, these natural oils are more readily available and industrially relevant [29].

**Fig. 6:**
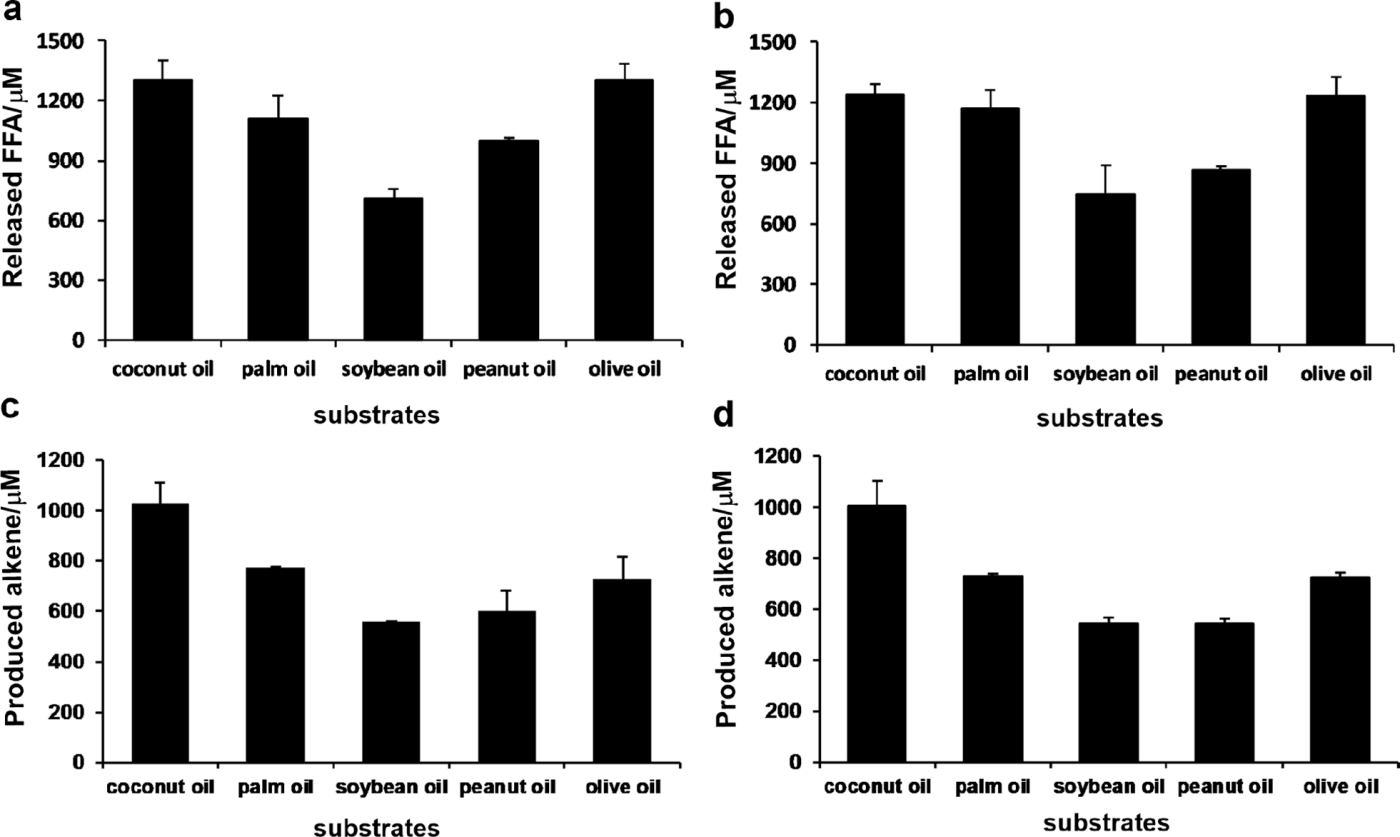
Released FFAs from hydrolysis of natural oils by CRL (**a**) and AOL (**b**); production of α-olefins from natural oils by the enzyme cascades mediated by CRL/OleT_JE_/AldO (**c**) and AOL/OleT_JE_/AldO (**d**)

In the CRL hydrolytic system, 1304.9, 1108.1, 708.1, 998.8, and 1301.9 μM of total FFAs were produced from 500 μM of coconut oil, palm oil, soybean oil, peanut oil, or olive oil, corresponding to 86.9%, 73.9%, 47.2%, 66.6%, and 86.8% of TAG-to-FFA conversions, respectively. Regarding the released FFA profiles of different natural oils (Additional file 1: Table S2), lauric acid, palmitic acid, and linoleic acid were the main hydrolytic products of coconut oil, palm oil, and soybean oil, respectively, accounting for 47.5%, 41.3%, and 54.7% of total FFAs. For both peanut oil and olive oil, oleic acid was the major released FFA species, reaching 45.6% and 71.9% of total FFAs, respectively. Moreover, the hydrolytic activities (Fig. 6a, b) and FFA profiles of the AOL hydrolytic system (Additional file 1: Table S3) toward the same group of natural oils were highly similar to those of the CRL hydrolytic system. Notably, coconut oil turned out to be the preferred substrate in both hydrolytic systems (Fig. 6a, b).

Subsequently, the one-pot enzyme cascade reactions including TAG hydrolysis, glycerol oxidation, and FFA decarboxylation using natural oils as starting materials were carried out. As results, 1027.5 (1002.9), 771.7 (731.3), 558.1 (544.4), 601.2 (543.7), and 730.7 μM (723.6 μM) of total α-olefins (including 1-heptene, 1-nonene, 1-undecene, 1-tridecene, 1-pentadecene, 1-heptadecene, and 1-nonadecene) were produced from coconut oil, palm oil, soybean oil, peanut oil, and olive oil, respectively (Fig. 6c, d, Additional file 1: Tables S4 and S5) by the CRL/AldO/OleT_JE_ (AOL/AldO/OleT_JE_) systems, corresponding to 68.5% (66.8%), 51.4% (48.8%), 37.2% (36.3%), 40.1% (36.2%), and 48.7% (48.2%) of TAG-to-alkene yields.

### Optimization of the coconut oil bioconversion system using the lyophilized CRL/OleT_JE_/AldO enzymes

Coconut oil among the tested natural oils appeared to be the best feedstock for *α*-olefin production (Fig. 6c, d), from which 1-undecene derived from lauric acid, the OleT_JE_-favored substrate, was the predominant product. Thus, the CRL/OleT_JE_/AldO system using coconut oil as the starting material to produce biological α-olefins was chosen for further optimization. To make the system more practical and cost-effective, lyophilized enzymes were used. Notably, the mixed lyophilized CRL, OleT_JE_, and AldO demonstrated comparable olefins-producing activity as the freshly purified proteins at the same levels (Additional file 1: Figure S3).

To examine the application potential of the one-pot reaction system consisting of CRL, OleT_JE_, AldO, coconut oil, and glycerol, the coconut oil concentration was increased from 500 to 1500 μM (~ 1 g/L). Under the higher substrate concentration, we re-optimized the amount of each individual component. As a result, 5 U CRL was shown to be able to hydrolyze 1500 μM coconut oil almost to the theoretical maximum yield of 4500 μM, and further increase of lipase amount had no significant beneficial effect on FFA production (Additional file 1: Figure S4a). However, when 5 U CRL, 3 μM OleT_JE_, and 15 μM AldO were reacted with 1500 μM coconut oil and 1.5% glycerol for 6 h, the total alkene yield was 14.3% lower than that of the previous 500 μM substrate system, indicating this reaction system needed to be further optimized.

Since 5 U CRL (among 2.5, 5, and 10 U) gave the highest alkene production (Additional file 1: Figure S4b), we subsequently optimized other contents in the TAG-to-olefin reaction system containing 5 U CRL. As expected, higher concentrations of OleT_JE_ and AldO resulted in improved alkene production under the same glycerol content (Table 2). Moreover, increase of glycerol content significantly improved the total yield of *α*-olefins. These results suggested that the H_2_O_2_ releasing upon glycerol oxidation might be the major limit of the overall efficiency of the alkene-producing system. Thus, the highest alkene yield achieved by this enzyme cascade system was 52.6% (0.53 g/L) at a 20% glycerol loading in a 200-μL reaction (Table 2). Of note, in a 10-mL reaction, this system delivered a similar *α*-olefin yield of 49.1% (0.49 g/L) (Table 2).

**Table 2:** α-Olefin production from 1500 μM coconut oil by CRL/OleT_JE_/AldO at 30 °C for 6 h under different reaction conditions

Enzyme concentrations	Glycerol content/%	Total α-olefins/μM	Yield/g/L^a^
3 μM OleT_JE_, 15 μM AldO	1.5	880.5 ± 1.7	0.20
	6	1377.9 ± 12.4	0.31
	10	1535.6 ± 82.2	0.34
	20	2138.3 ± 121.9	0.48
6 μM OleT_JE_, 30 μM AldO	3	1339.8 ± 137.4	0.30
	6	1416.6 ± 281.1	0.32
	10	1820.8 ± 239.5	0.41
	20	2369.9 ± 65.9	0.53
	20	2206.9 ± 27.9	0.49^b^

^a^200-μL reaction system

^b^10-mL reaction system. Error bars represent standard deviations derived from at least two independent experiments

## Discussion

With continuous discovery and characterization of novel hydrocarbon biosynthetic systems, a growing number of *α*-olefin-producing enzymes have been reported, including P450 fatty acid decarboxylases such as OleT_JE_ and CYP-Sm46 [11, 30], the non-heme iron-dependent decarboxylase UndA [31], and the membrane-bound desaturase-like UndB [32]. Although these *α*-olefin synthases can use different chain length FFAs as substrates, when they were coupled to the fatty acid biosynthetic systems of engineered *E. coli* or *Saccharomyces cerevisiae* strains, the total alkene titers turned out to be very low with the highest reported yield (97.6 mg/L) only accounting for about 1% of theoretical maximum yield (3% glucose) [18, 20, 32, 33]. Mechanistically, the complexity and tight regulation of fatty acid metabolic network in vivo could be one of the major reasons for the cost-prohibitive *α*-olefin yields [9].

To overcome this challenge, we previously engineered an in vitro tandem reaction system, in which the lipase Tll and OleT_JE_ cooperate to transform TAGs to α-olefins with exogenous addition of H_2_O_2_ [19]. Subsequently, Li et al. developed a genetically encoded synthetic self-assembled Tll/OleT_JE_ complex for bioproduction of fatty alkenes, wherein three non-catalytic modules including cohesion, dockerin, and CBM were used to control enzyme ratio, position, reusability, and stability [34]. However, the *α*-olefin yields of both approaches were low likely due to the detrimental effect of H_2_O_2_ for enzymes.

Although CYP152 peroxygenases are more H_2_O_2_-tolerant than most of P450 monooxygenases, a high concentration of H_2_O_2_ could still result in the loss of catalytic activity of these P450 peroxygenases, which was observed in the process of myristic acid hydroxylation catalyzed by the peroxygenase P450_CLA_ from *Clostridium actetobutylicum* [28, 35]. Despite the high activity (up to 200 min^−1^), substantial inactivation of P450_CLA_ occurred within 2–4 min when 200 μM H_2_O_2_ was added at once. Our results also showed that OleT_JE_ was plagued by the H_2_O_2_ concentrations greater than 1000 μM (Fig. 3). To resolve this problem, a growing number of approaches have been developed to elegantly control the H_2_O_2_ supply for reduction of the P450 inactivation [18, 36–38]. For example, a light-driven in situ H_2_O_2_-generating system employed flavin adenine mononucleotide (FMN) as a photocatalyst to reduce O_2_ to H_2_O_2_ with ethylenediaminetetraacetic acid (EDTA) as electron donor, which well supported OleT_JE_ to react with stearic acid. However, the conversion ratios of lauric acid and myristic acid were not satisfactory [38]. Moreover, redox partner engineering by making OleT_JE_-reductase fusion protein or constructing alternative OleT_JE_ reaction systems using separate redox partners has been proven as an effective method to reconstitute the decarboxylation activity of OleT_JE_ toward C_4_–C_22_ FFAs in the presence of NADPH regeneration system (e.g., formate/formate dehydrogenase, glucose/glucose oxidase) [18, 36, 37]. However, the requirement of expensive materials (FMN or NADPH) in both strategies and the complexity in the latter system consisting of four redox proteins would hinder their further application.

In this study, a novel enzyme cascade system was established to convert TAGs and natural oils into α-olefins in vitro (Fig. 1). Using the low-cost and renewable TAG/oil feedstock, the FFAs hydrolyzed off by lipases were well accepted by the downstream P450 fatty acid decarboxylase OleT_JE_. The required H_2_O_2_ cofactor of OleT_JE_ was continuously supplied in situ by the alditol oxidase AldO using the glycerol substrate derived from TAG hydrolysis, through which the H_2_O_2_-induced enzyme inactivation was significantly attenuated and the highest yield of α-olefins reached 0.53 g/L from 1500 μM coconut oil.

Although the enzyme cascade system provides a new paradigm for catalytically efficient and cost-effective biotransformation from TAGs/oils to α-olefins, the highest yield is still far from the stringent cost requirement of industrial production of α-olefins. There remain a number of significant problems to be overcome such as enzyme stability and solubility of hydrophobic substrates/products. It is anticipated that more enzyme engineering, process optimization, and other interdisciplinary approaches are required to make this enzyme cascade more industrially relevant.

## Conclusions

In this study, we established a new enzyme cascade independent of exogenous addition of H_2_O_2_, which is capable of efficiently converting a range of TAGs/natural oils into α-olefins. This one-pot biocatalytic system consisting of CRL (for TAG hydrolysis to provide FFAs and glycerol), AldO (for in situ H_2_O_2_ generation upon glycerol oxidation), and OleT_JE_ (for FFA decarboxylation using H_2_O_2_ as cofactor) was able to achieve a 68.5% total alkene yield from 500 μM coconut oil. Using the lyophilized enzymes, ~ 0.5 g/L of α-olefins were produced from the favorable feedstock coconut oil (1500 μM) upon some reaction optimization. Altogether, the three-enzyme cascade provides a new strategy for producing high value-added α-olefins from low-cost and renewable oils, demonstrating promising application potential.

## Methods

### Materials

The strains of *Escherichia coli* DH5α and BL21(DE3) and the plasmid pET28(b) were preserved by our laboratory. All antibiotics and chemicals including TAGs, FFAs, and α-olefins were obtained from Tokyo Chemical Industry (TCI) (Shanghai, China), Solarbio (Beijing, China), Sigma Aldrich (St. Louis, MO, USA), and Thermo Scientific (Shanghai, China). Soybean oil, peanut oil, and olive oil were purchased from local market. Coconut oil and palm oil were obtained from Orifera (Malaysia) and Pythonbio (Guangzhou, China), respectively. The 10 × QuickRun™ Fast Running Buffer and FlexiRun™ premixed gel solution for SDS-PAGE analysis were obtained from MDBio (Xinbei, China). Purification of DNA fragments was performed using a MonPure™ Gel & PCR Clean Kit from Monad (Wuhan, China). Ni–NTA resin used for protein purification was purchased from Sangon Biotech (Shanghai, China). PD-10 desalting columns were supplied by GE Healthcare (Piscataway, NJ, USA). Millipore Amicon Ultra centrifugal filters were bought from Millipore (Billerica, MA, USA).

### Molecular cloning and protein purification

The gene encoding AldO from *Streptomyces coelicolor* A3(2) (GenBank accession number: NC_003888.3) was codon-optimized and synthesized by Qinglan (Yixing, China), and then cloned into the vector pET28b via the *Nde*I/*Xho*I restriction sites for expression of the N-terminal His_6_-tagged recombinant proteins. The sequences of primers used in this study are listed in Additional file 1: Table S1. All cloned sequences were confirmed by DNA sequencing at Sangon Biotech (Shanghai, China), and then used to transform *E. coli* BL21 (DE3) for protein expression. The plasmid pET28b-*oleT*_*JE*_ for recombinant OleT_JE_ expression was constructed by this laboratory previously [18].

The *E. coli* BL21 (DE3) cells carrying a certain recombinant expression vector were grown at 37 °C for 12 h with shaking at 220 rpm and then used as seed cultures to inoculate (1:100 ratio) a modified Terrific Broth medium containing a rare salt solution [18]. Cells were grown at 37 °C for 3–4 h until the optical density at 600 nm (OD_600_) reached 0.8 to 1.0, to which 0.2 mM isopropyl β-d-1-thiogalactopyranoside (IPTG) was added. For P450 expression, 0.5 mM δ-aminolevulinic acid (5-ALA) and 1 mM thiamine were supplemented. Afterward, the cultivation continued for another 24 h at 18 °C for better protein folding [18, 26]. The cells were harvested (6000 ×* g*, 4 °C, 10 min) and stored at − 80 °C for later use.

Purification of the His-tagged OleT_JE_ was performed by following our previous procedure with some minor modifications [26]. Of note, the elution buffer (pH 7.4, 50 mM NaH_2_PO_4_, 500 mM NaCl) used in this study was glycerol free because glycerol is a reactant in the enzyme cascade reaction. Purification of the His-tagged AldO was carried out as described by Liu et al. with some modifications [18]. In brief, the cell pellets stored at − 80 °C were taken out to thaw at room temperature, then all the following steps were performed at 4 °C. The cells were re-suspended in 50 mL lysis buffer (pH 8.0, 50 mM NaH_2_PO_4_, 300 mM NaCl, 10 mM imidazole) through vortexing and then disrupted by ultra-sonication (SCIENTZ JY98-IIIDN, Ningbo; on 5 s/off 5 s for 30 min). Cell-free lysate was obtained by high-speed centrifugation (Eppendorf Centrifuge 5810R, Germany) at 10,000 × *g* for 1 h at 4 °C, to which 2 mL Ni–NTA resin slurry was added and mixed gently at 4 °C for 2–3 h. The mixture was loaded onto an empty column and washed with about 200 mL wash buffer (pH 8.0, 50 mM NaH_2_PO_4_, 300 mM NaCl, 20 mM imidazole) until no protein was detected in flow-through. The His-tagged proteins bound to Ni–NTA resin were eluted with 5–10 mL elution buffer (pH 8.0, 50 mM NaH_2_PO_4_, 500 mM NaCl, 250 mM imidazole). The eluents were concentrated with an Amicon Ultra centrifugal filter (30 kDa cutoff) and then buffer-exchanged into storage buffer (pH 8.0, 50 mM Tris–HCl). The solution containing purified proteins in aliquots were flash-frozen by liquid nitrogen for later use.

For preparation of the lipase Lip2, a single colony of *Yarrowia lipolytica* strain YLY [39] was inoculated into Yeast Extract Peptone Dextrose (YPD) medium for 12 h at 28 °C, and then used as seed cultures to inoculate (1:40 ratio) a fermentation medium [10 g yeast extract, 20 g tryptone, 10 g sucrose, and 6.7 g yeast nitrogen base (without amino acids) per liter], and shaking cultured at 28 °C, 220 rpm for 72 h. The supernatant containing secreted Lip2 was obtained by removing the cells by centrifugation (4 °C, 6000 × *g* for 10 min). The supernatant was concentrated with an Amicon Ultra centrifugal filter (30 kDa cutoff) and then buffer-exchanged into a Tris–HCl buffer (50 mM, pH = 8.0). The commercial lipases CRL and AOL were purchased from Sigma Aldrich (St. Louis, MO, USA).

### Enzyme concentration determination

Analysis of the UV–visible spectroscopic properties for OleT_JE_ was carried out as described previously [26]. The P450 protein concentration was calculated based on its reduced CO-bound difference spectrum using the reduced differential extinction coefficient ε_450–490_ of 91,000 M^−1^ cm^−1^ [18]. The concentration of AldO was determined at 452 nm with the reported extinction coefficient of 12,500 M^−1^ cm^−1^ [22]. The hydrolysis activity of lipase (U) was determined by the classical basic titration method using the olive oil emulsion. One hydrolysis activity unit (U) of lipase is defined as the amount of enzyme required to produce 1 μM free fatty acid in 1 min under the specific reaction conditions [40].

### In vitro enzymatic assay

Since P450 OleT_JE_ was characterized as a moderate halophilic protein requiring salt solution to maintain its stability and hence activity [26], all the reactions of OleT_JE_ were carried out in a buffer containing 500 mM NaCl. Although the high concentration of NaCl might negatively affect the activities of lipase and alditol oxidase, the compromise was made for the best performance of OleT_JE_.

A typical assay containing 1 μM OleT_JE_, 500/1000 μM lauric acid; 0–5000 μM H_2_O_2_, or 5 μM AldO and 0.01–10% *v/v* glycerol; and 5% EtOH as the co-solvent in 200 μL reaction buffer (pH 7.4, 50 mM NaH_2_PO_4_, 500 mM NaCl) was carried out at 30 °C for 6 h. The substrates including TAGs and natural oils were emulsified in water containing 2% (*w*/*w*) PVA [41] as emulsifier and dispersion stabilizer to prepare oil-in-water emulsions by ultra-sonication for better enzyme–substrate contacts.

For lipase-catalyzed hydrolysis, 5 U of lipase (CRL/AOL/Lip2), 500 μM TAGs (tricaprin, trilaurin, trimyristin, tripalmitin, or tristearin) or natural oils (coconut oil, palm oil, soybean oil, peanut oil, or olive oil) in 200 μL buffer (pH 7.4, 50 mM NaH_2_PO_4_, 500 mM NaCl) were mixed and incubated at 30 °C for 6 h.

For the one-pot reactions, 500 μM TAGs/natural oils, 5 U of lipase (CRL/AOL), 3 μM OleT_JE_, 15 μM AldO, 1.5% *v/v* glycerol, and 5% EtOH as the co-solvent in 200 μL buffer (pH 7.4, 50 mM NaH_2_PO_4_, 500 mM NaCl) were mixed and incubated at 30 °C for 6 h.

All reactions were quenched by adding 20 μL of 10 M HCl, then heptadecanoic acid (C_17_) was added as internal standard and the mixture was extracted by 150 μL ethyl acetate. The organic phase was analyzed by gas chromatography (GC) as described below. For detection of 1-heptene (C_7_) product generated from coconut oil in the enzyme cascade reactions, 1.5 mL polytetrafluorethylene (PTFE) septum-sealed glass bottles were used for the 200-μL reactions containing 3 μM OleT_JE_, 15 μM AldO, and 1.5% glycerol. The reactions were incubated at 30 °C for 6 h with shaking at 200 rpm. Then, the reactions were placed at 4 °C for 12 h to stop reactions prior to heating at 40 °C for 20 min for headspace sampling using a gas-tight Hamilton syringe for GC–MS analysis. Different concentrations of the authentic 1-heptene standard incubated under the same conditions of reactions were analyzed using the same GC–MS method to obtain the standard curve.

### Analytical methods

The hydrocarbon and fatty acid samples were analyzed by the methods modified from Guan et al. [42]. The Agilent 7890B gas chromatograph equipped with a capillary column HP-5 (Agilent Technologies, Santa Clara, CA, USA; 30 m × 0.32 mm × 0.25 μm) or HP-INNOWAX (Agilent Technologies, Inc, Santa Clara, CA, USA; 30 m × 0.25 mm × 0.25 μm) was used for analysis. The flow rate of helium was set to 1 mL min^-1^. The oven program was set initially at 40 °C for 5 min, then increased to 280 °C by the rate of 10 °C per min and held for 2 min. The injecting temperature was set to 280 °C under splitless injection conditions with 1 μL injection volume. The retention times and signal intensity of FAs and α-alkenes were determined and quantified with corresponding authentic standards (FAs: C_8_–C_20_, linoleic acid, linoleic acid; α-alkenes: C_7_–C_19_) and the internal standard [heptadecanoic acid (C_17_)]. For GC–MS analyses, the gas chromatography was equipped with an Agilent 5975C MSD single quadrupole mass spectrometer operated under electron ionization mode at 70 eV in the *m/z* scan range of 50 to 500 Da. The GC–MS analysis used the previous protocol adapted from Rude et al. [11] with the Agilent J&W DB-5MS column (30 m × 0.25 mm × 0.25 μm). Peak identity was determined by comparison of the retention time and fragmentation pattern with those of the authentic standard compounds that were available in the National Institute of Standards and Technology, USA mass spectral database.

## Supplementary information

**Additional file 1: Figure S1.** SDS-PAGE analysis of the secretory lipase Lip2 from *Yarrowia lipolytica* (lane A) and protein marker (M). **Figure S2.** SDS-PAGE analysis of the purified *N*-His_6_-tagged OleT_JE_ (lane A), *N*-His_6_-AldO (lane B), and protein marker (M). **Figure S3.** Comparison of α-olefin producing activities of freshly purified proteins and the lyophilized enzymes in the CRL-OleT_JE_-AldO tandem reaction system using 500 μM coconut oil as substrate. Error bars represent standard deviations derived from at least two independent experiments. **Figure S4.** (a) Total FFAs released from 1500 μM coconut oil by three different amounts of CRL; (b) the effect of CRL amount on the α-olefin production from 1500 μM coconut oil by the CRL/OleT_JE_/AldO system (3 μM OleT_JE_, 15 μM AldO, and 10% glycerol at 30 °C for 6 h). Error bars represent standard deviations derived from at least two independent experiments. Statistical analysis was performed using a Student’s *t* test (one-tailed; *P < 0.05, **P < 0.01, ns: *P *> 0.05, no significant; two-sample unequal variance). **Table S1.** Primers used in this study. **Table S2.** Released FFA profiles of different natural oils by lipase CRL. **Table S3.** Released FFA profiles of different natural oils by lipase AOL. **Table S4.** Distribution of *α*-olefins produced from natural oils by the tandem hydrolysis–oxidation-–decarboxylation reaction system of CRL/OleT_JE_/AldO. **Table S5.** Distribution of α-olefins produced from natural oils by the tandem hydrolysis–oxidation–decarboxylation reaction system of AOL/OleT_JE_/AldO.

## Data Availability

All data generated or analyzed during this study are included in this manuscript (and its Additional files).

## Abbreviations

CYP or P450: Cytochrome P450 enzyme
FFA: Free fatty acid
TAGs: Triglycerides
AldO: Alditol oxidase
CRL: Lipase from *Candida rugose*
AOL: Lipase from *Aspergillus oryzae*
Lip2: Lipase from *Yarrowia lipolytica*
TTN: Total turnover number
PCR: Polymerase chain reaction
PVA: Polyvinyl alcohol
GC–MS: Gas chromatography–mass spectrometry
