# Supplementary material for: Establishing an enzyme cascade for one-pot production of α-olefins from low-cost triglycerides and oils without exogenous H2O2 addition
Source: Biotechnol Biofuels. 2020 Mar 16;13:52. doi: 10.1186/s13068-020-01684-1 (PMC7075034; doi:10.1186/s13068-020-01684-1)
Supplement: Supplementary file 1 — Additional file 1: Figure S1. SDS-PAGE analysis of the secretory lipase Lip2 from Yarrowia lipolytica (lane A) and protein marker (M). Figure S2. SDS-PAGE analysis of the purified N-His6-tagged OleTJE (lane A), N-His6-AldO (lane B), and protein marker (M). Figure S3. Comparison of α-olefin producing activities of freshly purified proteins and the lyophilized enzymes in the CRL-OleTJE-AldO tandem reaction system using 500 μM coconut oil as substrate. Error bars represent standard deviations derived from at least two independent experiments. Figure S4. (a) Total FFAs released from 1500 μM coconut oil by three different amounts of CRL; (b) the effect of CRL amount on the α-olefin production from 1500 μM coconut oil by the CRL/OleTJE/AldO system (3 μM OleTJE, 15 μM AldO, and 10% glycerol at 30 °C for 6 h). Error bars represent standard deviations derived from at least two independent experiments. Statistical analysis was performed using a Student’s t test (one-tailed; *P < 0.05, **P < 0.01, ns: P > 0.05, no significant; two-sample unequal variance). Table S1. Primers used in this study. Table S2. Released FFA profiles of different natural oils by lipase CRL. Table S3. Released FFA profiles of different natural oils by lipase AOL. Table S4. Distribution of α-olefins produced from natural oils by the tandem hydrolysis–oxidation-–decarboxylation reaction system of CRL/OleTJE/AldO. Table S5. Distribution of α-olefins produced from natural oils by the tandem hydrolysis–oxidation–decarboxylation reaction system of AOL/OleTJE/AldO. [file 13068_2020_1684_MOESM1_ESM.pdf]

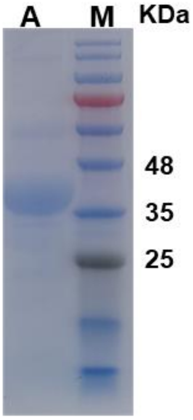

Figure S1. SDS-PAGE analysis of the secretory lipase Lip2 from *Yarrowia lipolytica* (lane A) and protein marker (M).

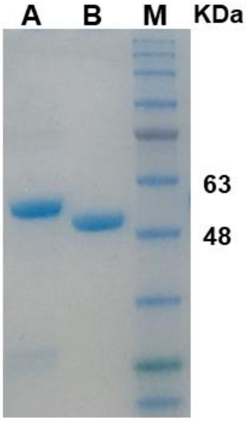

Figure S2. SDS-PAGE analysis of the purified *N*-His$_6$-tagged OleT$_{JE}$ (lane A), *N*-His$_6$-AldO (lane B), and protein marker (M).

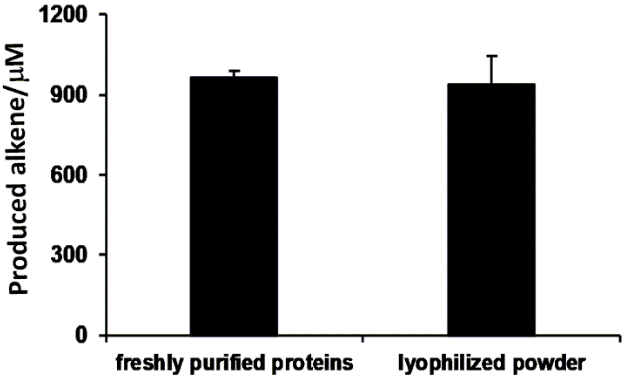

Figure S3. Comparison of α-olefin producing activities of freshly purified proteins and the lyophilized enzymes in the CRL-OleT$_{JE}$-AldO tandem reaction system using 500 μM coconut oil as substrate. Error bars represent standard deviations derived from at least two independent experiments.

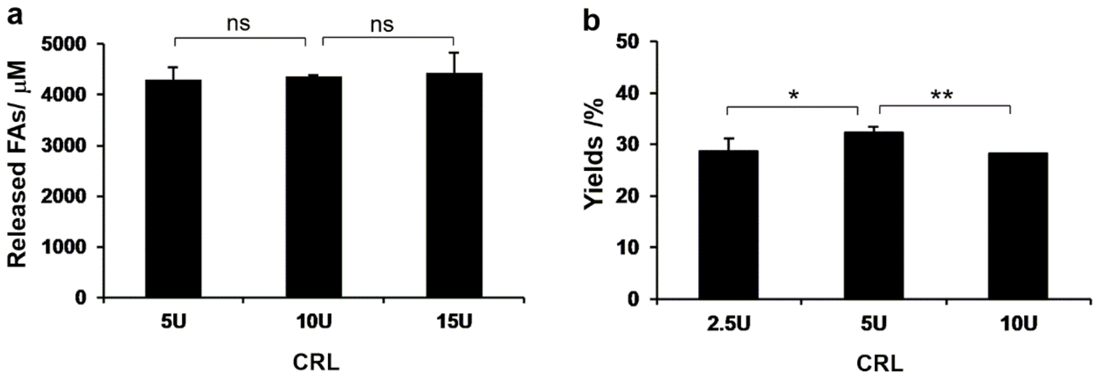

Figure S4. (a) Total FFAs released from 1500 μM coconut oil by three different amounts of CRL; (b) the effect of CRL amount on the α-olefin production from 1500 μM coconut oil by the CRL/OleT$_{JE}$/AldO system (3 μM OleT$_{JE}$, 15 μM AldO, and 10% glycerol at 30°C for 6 h). Error bars represent standard deviations derived from at least two independent experiments. Statistical analysis was performed using a Student's *t*-test (one-tailed; *P<0.05, **P<0.01, ns: P>0.05, no significant; two-sample unequal variance).

Table S1. Primers used in this study.

Primer	Sequence (5'→3')
OleT$_{JE}$-F	GGAATTC**CATATG**GCGACCCTGAAACGTGAT
OleT$_{JE}$-R	CCG**CTCGAG**TTAGGTGCGATCCACCACTTCG
AldO-F	GGAATTC**CATATG**AGCGATATTACCGTGACC
AldO-R	CCG**CTCGAG**TTAACCCGCTAACACGCCACGC

Note: The bold nucleotides denote the restriction site of *Nde*I or *Xho*I. Protective bases are shown in blue.

Table S2. Released FFA profiles of different natural oils by lipase CRL.

	Released FFAs/μM									
	C8	C10	C12	C14	C16	C18	C20	C16:1	C18:1	C18:2
Coconut oil	211.5 ± 18.5	122.8 ± 9.7	619.7 ± 43.9	173.9 ± 11.9	90.0 ± 7.2	34.1 ± 4.3	7.2 ± 2.4	0	45.7 ± 9.1	0
Palm oil	0	0	12.7 ± 9.5	17.6 ± 4.9	482.5 ± 54.7	53.9 ± 4.0	3.9 ± 0.2	20.4 ± 9.9	420.8 ± 12.8	96.2 ± 9.9
Soybean oil	0	0	0	0	110.7 ± 14.9	43.5 ± 1.5	3.2 ± 0.1	0	163.3 ± 32.1	387.3 ± 9.5
Peanut oil	0	0	0	0	130.2 ± 1.2	45.7 ± 0.7	9.9 ± 1.2	0	455.0 ± 8.3	357.9 ± 6.5
Olive oil	0	0	0	0	179.0 ± 14.1	44.9 ± 4.8	5.2 ± 0.5	16.5 ± 1.5	935.9 ± 55.0	120.3 ± 7.1

Note: Error bars represent standard deviations derived from at least two independent experiments.

Table S3. Released FFA profiles of different natural oils by lipase AOL.

	Released FFAs/μM									
	C8	C10	C12	C14	C16	C18	C20	C16:1	C18:1	C18:2
Coconut oil	175.6 ± 6.8	112.9 ± 5.0	592.1 ± 29.2	179.8 ± 13.2	87.1 ± 3.6	32.5 ± 2.0	7.2 ± 3.2	0	52.1 ± 6.6	0
Palm oil	0	0	9.9 ± 7.5	16.6 ± 3.7	508.2 ± 36.1	56.2 ± 2.2	3.8 ± 0.4	20.3 ± 9.8	423.1 ± 45.6	129.9 ± 14.0
Soybean oil	0	0	0	0	101.2 ± 22.8	40.3 ± 6.9	2.9 ± 0.3	0	170.3 ± 33.3	427.8 ± 83.6
Peanut oil	0	0	0	0	162.7 ± 3.9	56.2 ± 1.6	12.7 ± 0.8	0	376.5 ± 7.9	334.2 ± 6.9
Olive oil	0	0	0	0	162.6 ± 13.9	40.2 ± 2.9	4.4 ± 0.6	15.2 ± 1.3	893.4 ± 68.7	120.2 ± 9.2

Note: Error bars represent standard deviations derived from at least two independent experiments.

Table S4. Distribution of α-olefins produced from natural oils by the tandem hydrolysis-oxidation-decarboxylation reaction system of CRL/OleT$_{JE}$/AldO.

	Produced alkenes/μM								
	C7	C9	C11	C13	C15	C17	C19	C17:1	C17:2
coconut oil	153.3 ± 2.6	80.7 ± 1.4	496.2 ± 2.1	164.1 ± 1.3	53.1 ± 4.4	24.1 ± 0.1	0	45.1 ± 0.7	11.0 ± 0.1
palm oil	0	0	0	12.3 ± 0.7	264.2 ± 6.2	54.9 ± 1.1	0	330.3 ± 0.5	65.0 ± 1.0
soybean oil	0	0	0	0	73.5 ± 0.7	44.9 ± 1.7	0	170.5 ± 0.9	236.7 ± 1.6
peanut oil	0	0	0	0	78.0 ± 9.9	38.9 ± 5.3	0	282.5 ± 35.1	166.7 ± 21.3
olive oil	0	0	0	0	111.9 ± 7.2	44.0 ± 2.5	0	500.5 ± 64.3	31.6 ± 3.3

Note: Error bars represent standard deviations derived from at least two independent experiments.

Table S5. Distribution of α-olefins produced from natural oils by the tandem hydrolysis-oxidation-decarboxylation reaction system of AOL/OleT$_{JE}$/AldO.

	Produced alkenes/μM								
	C7	C9	C11	C13	C15	C17	C19	C17:1	C17:2
coconut oil	145.8 ± 40.5	96.2 ± 23.8	499.6 ± 39.4	168.7 ± 5.3	52.2 ± 1.1	22.5 ± 1.6	0	39.6 ± 5.3	11.1 ± 0.5
palm oil	0	0	0	11.7 ± 1.0	279.7 ± 43.2	55.8 ± 10.6	0	330.2 ± 56.7	59.4 ± 10.0
soybean oil	0	0	0	0	64.7 ± 3.2	36.9 ± 1.1	0	158.4 ± 7.5	213.3 ± 8.9
peanut oil	0	0	0	0	60.1 ± 2.7	30.2 ± 0.9	22.6 ± 2.9	229.5 ± 10.7	130.1 ± 7.3
olive oil	0	0	0	0	87.8 ± 10.3	26.3 ± 1.6	15.3 ± 0.8	481.9 ± 13.2	34.1 ± 0.8

Note: Error bars represent standard deviations derived from at least two independent experiments.

The codon-optimized gene sequence of *aldO*. *Nde*I and *Xho*I restriction sites are underlined. Start and stop codons are shown in red.

CATATGAGCGATATTACCGTGACCAACTGGGCGGGCAACATTACCTATACCGCGAAAGA
ACTGTTACGCCCGCATAGCCTGGATGCATTACGTGCGCTGGTTGCGGATAGCGCACGTG
TTCGCGTTTTAGGTAGCGGCCATAGCTTTAACGAAATTGCGGAACCGGGTGATGGTGG
CGTTTTATTAAGCCTGGCGGGCTTACCTAGCGTGGTTGATGTGGATACCGCGGCACGTA
CCGTTCGTGTTGGTGGCGGCGTTCGTTATGCGGAATTAGCGCGCGTTGTTCATGCGCGT
GGCTTAGCGTTACCTAACATGGCGAGCCTGCCGCATATTAGCGTGGCAGGTAGCGTTGC
GACTGGTACCCATGGTAGCGGTGTTGGTAACGGCTCACTGGCGAGCGTTGTGCGTGAA
GTGGAACTGGTTACCGCAGATGGCAGCACCGTTGTTATTGCGCGTGGCGATGAACGTT
TTGGCGGTGCGGTTACCAGCTTAGGTGCGTTAGGCGTTGTGACCAGCTTAACCCTGGA
TCTGGAACCGGCGTATGAAATGGAACAGCACGTGTTTACCGAATTACCGCTGGCGGGT
TTAGATCCGGCGACCTTTGAAACCGTTATGGCGGCGGCGTATAGCGTTAGCCTGTTTAC
CGATTGGCGCGCGCCTGGTTTTCGTCAAGTGTGGCTGAAACGCCGTACCGATCGTCCT
CTGGATGGCTTTCCTTATGCGGCACCGGCGGCGGAAAAAATGCATCCGGTGCCTGGTAT
GCCTGCAGTTAACTGCACCGAACAGTTTGGCGTTCCTGGCCCTTGGCATGAACGCTTA
CCGCATTTTCGCGCGGAATTTACCCCTAGCAGCGGTGCGGAACTGCAGAGCGAATATCT
GATGCCGCGCGAACATGCGTTAGCGGCGTTACATGCGATGGATGCGATTCGTGAAACC
CTGGCGCCTGTGTTACAGACCTGCGAAATTCGTACCGTTGCGGCGGATGCGCAATGGC
TGAGCCCTGCGTATGGTCGTGATACCGTGGCGGCACATTTTACCTGGGTGGAAGATACC
GCGGCGGTTTTACCTGTTGTGCGCCGTCTGGAAGAAGCGTTAGTGCCTTTTGCGGCGC
GTCCTCATTGGGGCAAAGTGTTTACCGTTCCGGCGGGTGAATTACGCGCGTTATATCCG
CGTCTGGCGGATTTTGGTGCATTAGCGGGTGCGTTAGATCCTGCGGGCAAATTTACCAA
CGCGTTTGTGCGTGGCGTGTTAGCGGGTTAACTCGAG
